# Spinal Epidermoid Tumor: A Case Report

**DOI:** 10.7759/cureus.36409

**Published:** 2023-03-20

**Authors:** Joseph Yunga Tigre, Meredith Costello, Krisna Maddy, Emily Errante, Stephen S Burks

**Affiliations:** 1 Department of Neurosurgery, University of Miami Miller School of Medicine, Miami, USA

**Keywords:** epidermoid cyst, spinal cord diseases, intraspinal tumor, spinal tumor, epidermoid tumor

## Abstract

Spinal epidermoid tumors are rare tumors with typical symptoms including low back pain, radiculopathy, weakness, sensory disturbances, and bowel/bladder dysfunction. Here we present a rare case of a spinal epidermoid tumor in a 44-year-old female patient with a previous surgical history of epidural anesthesia with two cesarean sections. Our report aims to highlight the rare development of this type of tumor following epidural anesthesia, a routine part of labor management.

## Introduction

Spinal epidermoid tumors, which make up less than 1% of intraspinal tumors, are composed of epidermal cells implanted in the spinal canal [1]. This implantation can occur following trauma, surgery, or be present as a congenital anomaly [1]. Patients with a history of lumbar punctures are at an increased risk for developing these types of tumors [2]. Here we present a rare case of a spinal epidermoid tumor in a 44-year-old female patient with a previous history of epidural anesthesia for cesarean section that resulted in a dural injury.

## Case presentation

Initial presentation

**Figure 1:**
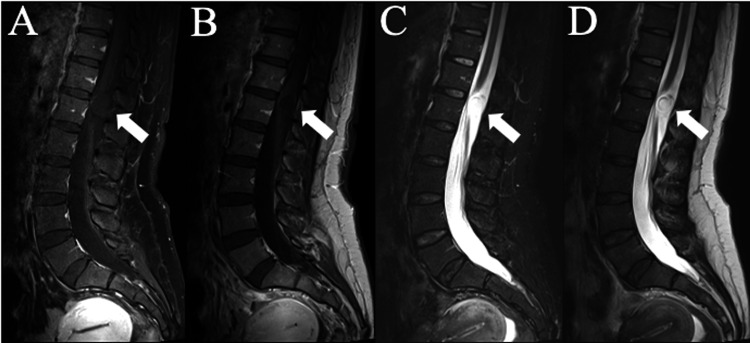
Sagittal thoracic and lumbar MRIs demonstrating T12-L1 intradural extramedullary lesion The sagittal thoracic and lumbar spine MRIs demonstrate the T12-L1 lesion (white arrow) found on imaging. (A) is a sagittal T1 MRI of the thoracic and lumbar spine with gadolinium. (B) is a sagittal T1 MRI of the thoracic and lumbar spine without gadolinium. (C) is a sagittal T2 STIR MRI of the thoracic and lumbar spine. (D) is a sagittal T2 MRI of the thoracic and lumbar spine.

**Figure 2:**
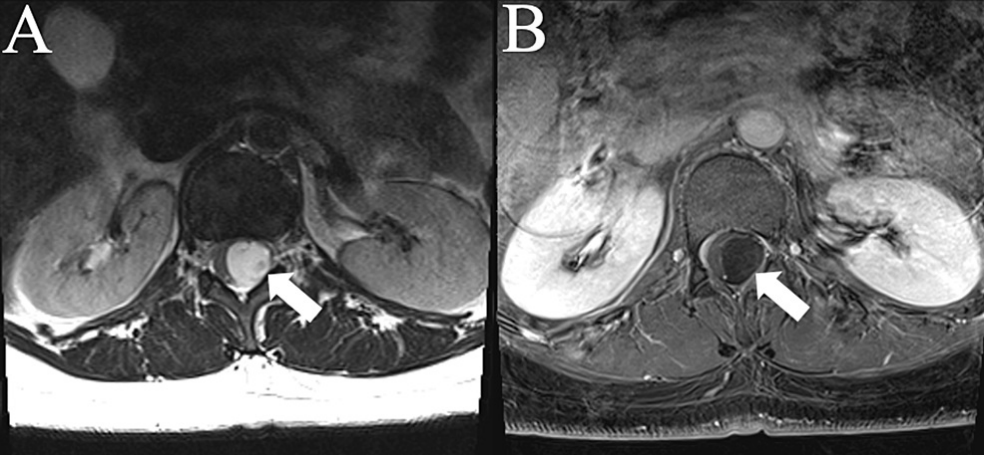
Axial MRIs demonstrating the T12-L1 intradural extramedullary lesion The axial MRIs demonstrate the T12-L1 lesion (white arrow) found on imaging. (A) is an axial T2 MRI without gadolinium. (B) is an axial T2 MRI with gadolinium, showing no evidence of enhancement.

A 44-year-old right-handed female with a past medical history of low back pain and left leg numbness presented for evaluation. She reported back pain that began five years ago but experienced new onset left leg numbness and paresthesia six months ago. She described the back pain as intermittent and worse with prolonged sitting, bending, or lifting. She also reported occasional shocking sensations down the left posterior leg, as well as weakness of the left leg. She denied bowel/bladder incontinence or balance difficulties. However, she did endorse a distance-limited gait due to her left leg pain. Analgesics previously improved her back pain, but lately she found no improvement. Her surgical history was significant for a previous right axilla cyst removal for hidradenitis, laparoscopic fulguration for endometriosis, and two previous cesarean sections. Of note, the patient reported the development of a spinal headache after her second C-section requiring bedrest and an epidural blood patch. These complaints were suggestive of a dural injury. Her physical examination was unremarkable, with a full range of motion of the cervical and lumbar spine with no pain elicited on movement. She had full strength in both legs with slightly diminished sensation to light touch in the left leg and foot in a non-dermatomal distribution. Reflexes were elevated at the lower extremities bilaterally, but no clonus or Babinski sign was detected. MRIs were obtained, and she was found to have an intradural extramedullary lesion at the T12-L1 level with severe deformation of the spinal cord (Figures 1-2). 

Intervention

**Figure 3:**
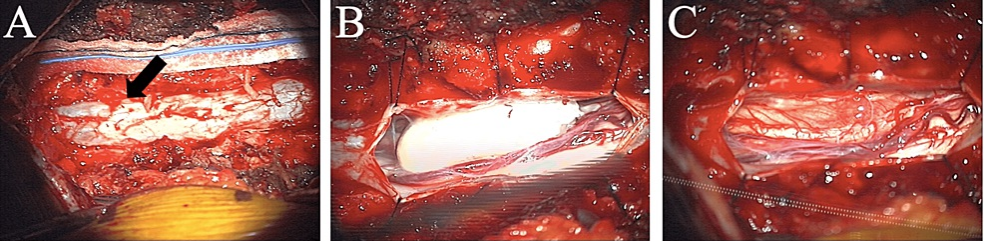
Surgical exposure of lesion and subsequent resection The intraoperative images show the surgical exposure and resection of the lesion. (A) portrays the small outpouching of the dura, with the black arrow demonstrating the dural dehiscence noted upon exposure. (B) demonstrates the exposed pearly white tumor. (C) shows total gross resection of the tumor.

**Figure 4:**
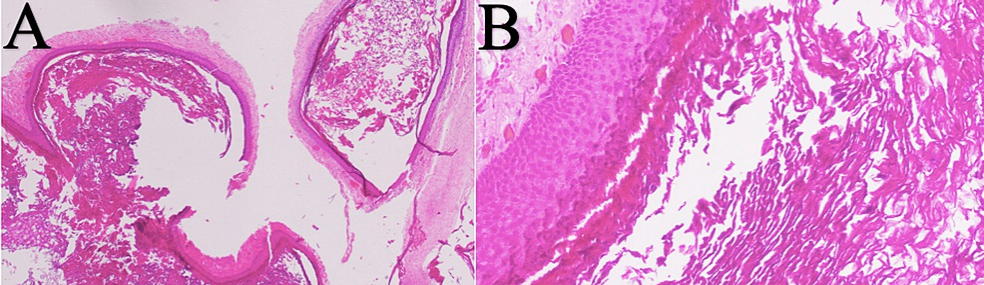
Histopathologic slides of the lesion consistent with an epidermoid tumor The histopathologic images of the lesion are shown above. (A) and (B) are images of the histopathologic slides of the lesion consistent with an epidermoid tumor.

After a discussion with the patient and her husband, a mutual decision was made to proceed with T12 to L1 laminectomy with intradural exploration and removal of an intradural extramedullary tumor. On the day of surgery, the patient was carefully intubated and positioned on the Jackson table. Intraoperative neuromonitoring with motor evoked potentials (MEPs) and spinal somatosensory evoked potentials (SSEPs) were used throughout the surgery. A laminectomy was performed, including complete removal of T12 and L1. The dura was inspected and immediately caudal to the L1 lamina (L1-2 interspace) a small outpouching on the dura was noted (Figure 3A). This possibly indicated the site of the prior dural injury from attempted epidural injection. An ultrasound was performed that confirmed the exposure extended above and below the intradural pathology. The dura was then incised and opened using the operating microscope. The dural leaflets were tacked up, and a pearly, white tumor was seen (Figure 3B). While maintaining a layer of arachnoid between the tumor and the nerves, gross total resection of the tumor was achieved (Figure 3C). The dura and wound were closed at this point, and the patient was transferred back to the Post-Anesthesia Care Unit (PACU). The final pathology review confirmed the lesion as an epidermoid tumor (Figure 4).

## Discussion

Spinal epidermoid tumors are rare and are more commonly seen in children [1]. Based on a literature review conducted by Park et al., they found only three cases of epidermoid tumors in adults older than 30 years old [1]. Spinal epidermoid tumors can have a wide-ranging clinical presentation depending on the location of the tumor [2]. Typical symptoms include low back pain, radiculopathy, weakness, sensory disturbances, and bowel/bladder dysfunction [2]. In a study reviewing seven cases of epidermoid tumors by Mortia et al., they found that the average time to the clinical presentation from the inciting event for epidermoid tumors was nine years [2]. Our patient presented with symptoms consistent with those of a spinal epidermoid tumor, including low back pain, radiculopathy, and sensory disturbance. Upon detailed questioning of the patient, as noted above, she described symptoms consistent with a cerebrospinal fluid (CSF) leak following her second cesarian section. She recalls being placed on bedrest and ultimately requiring an epidural blood patch due to severe positional headache. On exposure of the dura, a small outpouching was identified. This appeared to represent an area of prior dural injury but was oddly located at the L1-2 level. This might be due to the patient’s concomitant diagnosis of thoracolumbar scoliosis, making the lumbar puncture more difficult. Unfortunately, medical records from her prior hospitalizations are not available, and these hospitalizations were outside of the US.

Epidural injections are commonly used in labor for pain management. In a review studying labor pain treatment options by Koyyalamudi et al., they reported approximately 61% of women in the USA received neuraxial analgesia for labor pain management [3]. Similarly, Anim-Somauh et al. report that the use of epidural analgesia in labor has increased over the past 20 years, with more than 60% of women in the USA, 20% of women in the UK, and an increasing number of women in China using this option for pain management in labor [4].

Our case exhibits the development of a rare complication from dural injury related to an epidural injection, an epidermoid tumor developing years after the procedure. Such a complication is not likely to be considered when performing this procedure. This inciting event may be overlooked as a spinal intervention, as it is a routine part of labor or pain management. In a case series evaluated by Mortia et al., they found that three out of four patients who developed an epidermoid tumor had prior lumbar punctures performed [5]. In addition, our case highlights the rare complication of epidermoid tumor development in a similar situation. Previous studies have reported the association between epidermoid tumors and lumbar punctures; however, this association is seen in lumbar punctures performed in early childhood [6] and less often in adulthood.

## Conclusions

Spinal epidermoid tumors are rare and sparsely reported on in the current literature. We present a case of an epidermoid tumor in a female patient with a history of epidural injection associated with a spinal fluid leak. We show an abnormality on the dura likely associated with the site of prior intervention and in close proximity to the epidermoid tumor.
